# The 2026 Bundibugyo virus disease outbreak in the Democratic Republic of the Congo: Virology, epidemiology, continental response, and research priorities

**DOI:** 10.1016/j.imj.2026.100270

**Published:** 2026-06-25

**Authors:** Yi Zhang, Junkai Ren, Xuejun Ma, Xiaozhou He

**Affiliations:** National Key Laboratory of Intelligent Tracking and Forecasting for Infectious Diseases, National Institute for Viral Disease Control and Prevention, Chinese Center for Disease Control and Prevention, Beijing, 102206, China

**Keywords:** Ebola virus, Bundibugyo virus disease, Public health emergency of international concern, Continental response, Vaccine development, Non-pharmaceutical intervention

## Abstract

*Bundibugyo ebolavirus* (BDBV), a member of the genus *Orthoebolavirus*, family *Filoviridae*, is hereafter referred to as Bundibugyo virus, with the associated illness termed Bundibugyo virus disease (BVD). First identified in Uganda in 2007, the virus has received far less scientific attention than the highly pathogenic *Zaire ebolavirus*. In April 2026, a cluster of unexplained deaths emerged in the mining-intensive Mongbwalu Health Zone of Ituri Province in the Democratic Republic of the Congo (DRC), and the causative agent was confirmed by whole-genome sequencing on 14 May 2026 as Bundibugyo virus. This is the DRC’s 17th Ebola outbreak and only the third BVD epidemic globally. As of 10 June 2026, the DRC has reported 662 confirmed cases and 124 confirmed deaths, with the outbreak expanding across 25 health zones in Ituri, North Kivu, and South Kivu provinces; Uganda has reported 19 confirmed cases and 2 deaths. Unlike *Zaire ebolavirus* outbreaks, no licensed vaccine or specific therapeutic exists for BVD, and the response is further complicated by armed conflict, population mobility through mining corridors, fragile health systems, and community mistrust that has occasionally erupted into violence against responders. The World Health Organization (WHO) declared a Public Health Emergency of International Concern (PHEIC) on 17 May 2026, and the Africa Centres for Disease Control and Prevention (Africa CDC) declared a Public Health Emergency of Continental Security (PHECS) on 18 May 2026. Under a unified “One Team, One Plan, One Budget, and One M&E Framework” architecture, the Africa Centres for Disease Control and Prevention and World Health Organization jointly issued the *Bundibugyo Virus Disease Continental Preparedness and Response Plan*, with a budget of $517.7 million over six months and structured around 14 technical pillars. This review provides a comprehensive synthesis of the virology, epidemiology, critical research gaps, and international response strategy, aiming to inform both ongoing outbreak operations and future scientific efforts.

## 1 Introduction

Ebola virus disease is among the deadliest infectious diseases known to humanity.^1^, ^2^, ^3^ Of the six recognized ebolavirus species, *Zaire ebolavirus* has attracted the greatest research investment because of its role in the 2013–2016 West African epidemic.^4^^,^^5^ In sharp contrast, *Bundibugyo ebolavirus* had been documented in only two prior outbreaks—a 2007 epidemic in Uganda (131 suspected cases, 42 deaths) and a 2012 outbreak in the Democratic Republic of the Congo (DRC) (77 suspected cases, 36 deaths).^6^^,^^7^—leaving its scientific understanding and medical countermeasure (MCM) pipeline severely underdeveloped.

Between April and May 2026, eastern DRC became the epicenter of the third Bundibugyo virus disease (BVD) epidemic in history.^8^^,^^9^ The outbreak showed several alarming features: Origin in a mining zone, a silent transmission window of approximately five weeks before confirmation, expansion from Ituri into North and South Kivu provinces, cross-border importation into Uganda, healthcare worker (HCW) infections, and attacks on safe burial teams.^9^ World Health Organization (WHO) declared a Public Health Emergency of Continental Security (PHEIC) on 17 May.^10^, and Africa Centres for Disease Control and Prevention (Africa CDC) declared a PHECS on 18 May.^11^; Africa CDC subsequently noted that this had become the largest Ebola outbreak in Africa since the 2014–2016 West African crisis.^9^

This review systematically examines BVD virology, historical and current epidemiology, the 14-pillar Africa CDC–WHO joint continental response strategy, priority research gaps, and forward-looking recommendations for scientific prevention, control, and diagnostic development.

## 2 Virology

### 2.1 Taxonomy and genome

The genus *Orthoebolavirus* comprises six recognized species.^12^^,^^13^: *Zaire ebolavirus, Sudan ebolavirus, Bundibugyo ebolavirus, Taï Forest ebolavirus, Reston ebolavirus*, and *Bombali ebolavirus*. Among these, the first four are known to cause disease in humans; *Reston ebolavirus* is pathogenic in nonhuman primates and pigs but has not been associated with human illness, while *Bombali ebolavirus* has been identified exclusively in bats and its pathogenic potential for humans remains uncharacterized.^13^ *Bundibugyo ebolavirus* is the fourth species in the genus confirmed to cause human disease.^12^^,^^13^ The International Committee on Taxonomy of Viruses (ICTV) 2019 revision recognizes six species in this genus.^14^ The genome is a single-stranded negative-sense RNA of approximately 18.9 kb, encoding seven structural proteins.^15^^,^^16^ The first complete sequence (GenBank NC_014373) was published in 2008.^17^ Compared with *Zaire ebolavirus*, nucleotide and amino acid divergence in the GP gene is about 32% and 37%, respectively.^18^—a distance that fundamentally compromises the cross-protective potential of existing Zaire-based vaccines and antibody therapeutics.

### 2.2 Reservoir and ecology

Fruit bats of the family Pteropodidae are considered the natural reservoir for filoviruses, though conclusive virus isolation is lacking.^19^^,^^20^ Serological evidence exists in bats in Uganda and eastern DRC.^21^ but the exact reservoir host(s) and spillover conditions for *Bundibugyo ebolavirus* (BDBV) remain undefined. The 2026 outbreak’s origin in a mining zone suggests that ecological disturbance of bat habitats may have increased human exposure; however, the specific spillover event has not been identified.^9^

### 2.3 Pathogenicity and prior presentations

BDBV is a BSL-4 pathogen.^22^ The 2007 outbreak had a case fatality ratio (CFR) of ∼ 25%.^6^^,^^23^; the 2012 outbreak ∼ 36%.^7^ Both exhibited classic Ebola syndromes: Acute fever, weakness, vomiting, diarrhea, and hemorrhage in 40%–50% of patients.^24^

As of 10 June 2026, the case fatality ratio among confirmed cases stands at approximately 18.5%, likely biased by under-ascertainment of community deaths, lack of laboratory confirmation for many suspected cases, and the possible existence of mild or asymptomatic infections.^25^ Co-circulation of malaria, arboviruses, and typhoid masked early clinical recognition.^9^

### 2.4 Contrast with *Zaire ebolavirus* and MCM gaps

The most operationally critical difference is the complete absence of licensed MCMs for BDBV. WHO-recommended monoclonal antibodies and the rVSV-ZEBOV vaccine were all validated on EBOV GP.^26^^,^^27^ and offer little cross-protection against BDBV.^28^^,^^29^ The initial local testing at Bunia used a *Zaire*-specific GeneXpert cartridge and returned a false-negative result, delaying confirmation for several days until pan-filovirus testing at the Institut National de Recherche Biomédicale (INRB) in Kinshasa identified the pathogen.^9^ While certain broadly reactive antibodies have shown in vitro cross-neutralization.^30^, none are near clinical evaluation. WHO and Africa CDC have activated the interim Medical Countermeasures Network (i-MCM-net), engaging Gavi, the Coalition for Epidemic Preparedness Innovations (CEPI), and International Drug Purchase Facility (UNITAID) to accelerate candidate evaluation.^12^

## 3 Epidemiology

### 3.1 Prior outbreaks

The 2007 Uganda outbreak (131 suspected cases, 42 deaths).^6^^,^^31^ and the 2012 DRC outbreak (77 suspected cases, 36 deaths).^7^ displayed > 98% genomic identity, suggesting persistent circulation or repeated spillover in the region.^32^

### 3.2 Timeline and geographic spread of the 2026 outbreak

**Fig. 1:**
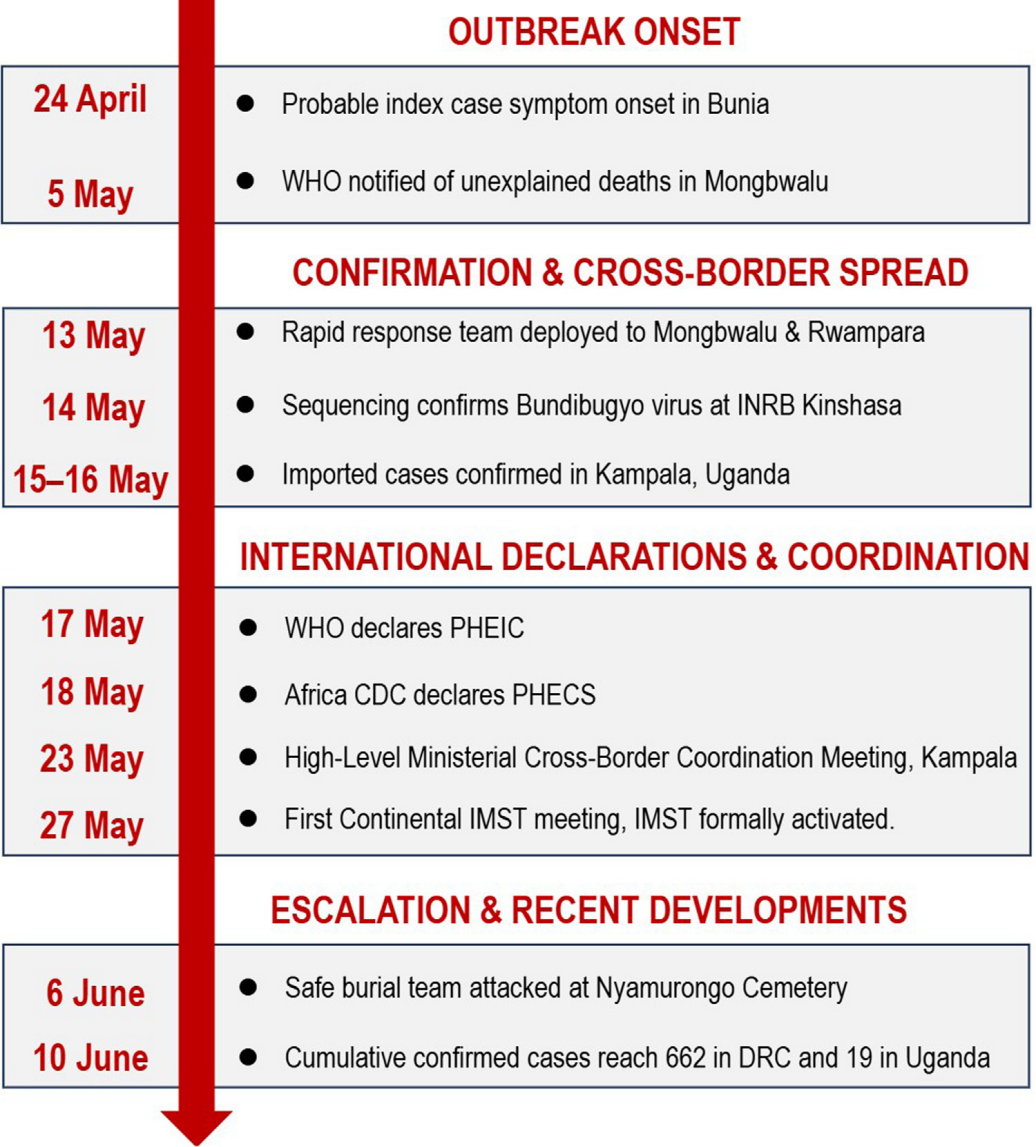
Chronology of major epidemiological, laboratory, and operational milestones in the 2026 Bundibugyo virus disease outbreak. Events span from initial case detection (24 April 2026) through 10 June 2026 and include international declarations (PHEIC, PHECS), cross-border importation to Uganda. *Abbreviation:* Africa CDC, Africa Centers for Disease Control and Prevention; DRC, Democratic Republic of the Congo; INRB, Institut National de Recherche Biomédicale; PHECS, Public Health Emergency of Continental Security; PHEIC, Public Health Emergency of International Concern; WHO, World Health Organization.

A comprehensive timeline of key events in the 2026 outbreak is presented in Fig. 1. The probable index case, a volunteer health worker, developed symptoms on 24 April 2026 in Bunia and died shortly thereafter. WHO was notified on 5 May of unexplained deaths in Mongbwalu, where four health workers died within four days. The GeneXpert *Zaire*-specific assay at Bunia was negative; samples sent to INRB Kinshasa tested positive on a pan-filovirus assay, and whole-genome sequencing confirmed BDBV on 14 May 2026. Several weeks of undetected transmission were documented between epidemiological weeks 15 and 19.^9^

On 15–16 May, two laboratory-confirmed cases (one death) were reported at Kibuli Hospital in Kampala, Uganda, linked to travel from Bunia.^9^ Uganda activated its National Task Force on 16 May. As of 7 June, Uganda had 19 confirmed cases, 2 confirmed deaths, and 5 HCW infections.^25^

As of 27 May, the outbreak had reached 15 health zones across Ituri, North Kivu (Butembo, Katwa, Goma), and South Kivu (Miti-Murhesa).^9^; by 7 June, the number rose to 25 health zones in DRC.^25^ Bunia’s role as a regional transport and trade hub amplifies regional spread risk.^9^

### 3.3 Transmission dynamics

Formal R₀ estimates have not been published, but a 4–5 week silent window, syndromic masking, high mining-related mobility, and infection prevention and control (IPC) scores as low as 7% and 34%.^9^ indicate elevated transmission potential. By 10 June, 6525 contacts had been listed in the DRC, with only 76% under follow-up, constrained by conflict.^25^ HCW infections rose from an initial cluster of 4 to 29 (5 deaths).^9^^,^^25^

### 3.4 Compounding drivers

Decades of armed conflict in Ituri restrict team deployment, specimen transport, and supply delivery.^9^^,^^33^ On 6 June, a safe and dignified burial (SDB) team was attacked at Nyamurongo Cemetery, leaving two responders badly injured and two vehicles damaged.^25^

Mongbwalu’s artisanal gold mines attract informal miners from Uganda and South Sudan.^34^, generating efficient cross-border transmission corridors along porous border crossings. The Uganda Martyrs’ Day pilgrimage on 3 June was flagged as a high-risk mass gathering.^9^

Health infrastructure is fragile, with shortages of isolation beds, personal protective equipment (PPE), and laboratory capacity.^9^ On 7 June, a sharp decline in outpatient attendance was reported at Bunia General Referral Hospital, driven by community fear of nosocomial contamination; Human Immunodeficiency Virus (HIV), Tuberculosis (TB), Non Communicable Diseases (NCD) and antenatal visits were widely missed without a catch-up plan.^25^

Community mistrust, echoing dynamics from the 2018 EBOV epidemic in eastern DRC.^35^, has led to delayed healthcare seeking, resistance to public health measures, and attacks on response teams. The continental plan embedded community-centered and psychosocially informed response as a guiding principle.^9^

### 3.5 Risk assessment and categorization

Africa CDC and WHO assess the national risk for DRC as very high, regional risk as high, and global risk as low.^9^ Africa Union (AU) Member States are prioritized into four tiers: Priority 1A (DRC, Uganda) for full multi-pillar response; Priority 1B (South Sudan, Rwanda, Burundi) for operational readiness and cross-border coordination; Priority 2 (8 countries) for minimum operational requirements; and Priority 3 for the remainder.^9^ The downgrading benchmark is 42 consecutive days without a new confirmed or probable case, followed by at least six months of enhanced surveillance.^9^^,^^36^

Although the global risk is currently assessed as low, the detection of imported cases in Kampala—a city with an international airport and travel connections to Europe, the Middle East, and Asia—raises the possibility of limited intercontinental spread. Experience from the 2013–2016 West African epidemic demonstrated that sporadic exportation of Ebola cases to non-endemic countries via commercial air travel can occur, though sustained transmission in settings with robust health systems is unlikely.^10^^,^^36^ The current outbreak therefore warrants enhanced vigilance at international points of entry and the pre-positioning of pan-filovirus diagnostic capacity in reference laboratories worldwide.

## 4 Medical countermeasures and research gaps

### 4.1 Vaccines

Licensed *Zaire ebolavirus* vaccines—rVSV- CEPI, ChAd3-EBO-Z, and Ad26.ZEBOV/MVA-BN-Filo—rely on EBOV GP and are unlikely to confer cross-protection against BDBV.^27^^,^^37^^,^^38^ Several BDBV-specific candidates based on VSV, chimpanzee adenovirus, DNA, and virus like particle (VLP) platforms are in preclinical stages.^39^, ^40^, ^41^, ^42^; certain multivalent candidates have shown partial protection in nonhuman primates.^43^ The continental plan calls for outbreak-ready clinical trial protocols and adaptive designs to accelerate evaluation.^9^, with coordination through i-MCM-net.^9^

### 4.2 Therapeutics

The WHO-recommended EBOV monoclonal antibodies have minimal cross-neutralizing activity against BDBV.^29^^,^^44^ Broadly reactive filovirus monoclonal antibodies have shown partial cross-neutralization in vitro.^30^ Small-molecule antivirals such as remdesivir and galidesivir, which target the polymerase, theoretically have broad-spectrum activity but lack in vivo data against BDBV.^45^; other investigational compounds include FGI-106.^46^

### 4.3 Diagnostics

The false-negative result from the *Zaire*-specific GeneXpert cartridge at the Bunia laboratory exposed a critical diagnostic vulnerability.^9^ Most prequalified molecular assays target *Zaire ebolavirus* conserved regions, and primer/probe mismatches with BDBV can reduce sensitivity.^47^ No BDBV-specific rapid antigen test has been prequalified by WHO. Multiplex pan-filovirus assays, Clustered Regularly Interspaced Short Palindromic Repeats (CRISPR)-based platforms.^48^^,^^49^, and expanded genomic sequencing networks.^50^^,^^51^ are priority development areas.

### 4.4 Non-pharmaceutical interventions and implementation science

IPC, safe burials, contact tracing, and community engagement collectively account for ∼ 37% of the response budget and form the backbone of containment in the absence of MCMs.^9^ However, the evidence base for implementing non-pharmaceutical interventions (NPIs) in conflict-affected, low-trust settings is weak.^52^^,^^53^ The attack on an SDB team on 6 June and the collapse of hospital attendance in Bunia.^25^ illustrate the implementation challenges. The plan’s innovative approaches—co-designing alternative mourning rituals with communities, engaging traditional healers as partners—require rigorous operational evaluation.

### 4.5 Viral persistence and survivor care

While EBOV is known to persist in immune-privileged sites with documented sexual transmission^54^, ^55^, ^56^, ^57^, BDBV’s tissue tropism and persistence duration are unknown.^58^ The continental plan mandates a dedicated survivor care unit for motor, pain, vision, and cognitive impairments. With only 20 recoveries recorded across both countries as of 7 June.^25^, the survivor cohort remains small, and long-term follow-up systems need to be established urgently.

### 4.6 Viral ecology and One Health

The mining-area origin underscores the importance of investigating ecological disturbance as a driver of spillover.^35^ The animal reservoir and ecological dynamics of BDBV are virtually uncharacterized.^19^ The plan’s standalone One Health pillar (Pillar 12) calls for joint human–animal–environmental risk assessments and surveillance at high-risk interfaces such as mining sites.^9^

## 5 International response: The joint Africa CDC–WHO strategy

### 5.1 One Team, One Plan, One Budget architecture

On 22 May 2026, Africa CDC and WHO adopted a unified “One Response” approach and activated the Continental Incident Management Support Team (IMST).^9^ The first IMST strategic partnership meeting was held on 27 May.

A high-level ministerial meeting convened in Kampala on 23 May, addressed by President Ndayishimiye, President Ramaphosa, Chairperson Youssouf, Dr. Kaseya, and Professor Janabi.^9^ President Ramaphosa noted that Africa CDC had declared this the second-largest Ebola epidemic after the 2014–2016 West African crisis and announced a $5 million contribution from South Africa.^12^

### 5.2 The continental preparedness and response plan

The *Bundibugyo Virus Disease Continental Preparedness and Response Plan* (June–November 2026) was endorsed at the Kampala meeting, with a budget of $517,678,605 and structured around 14 technical pillars.^9^ Core areas include: Coordination, leadership and governance—establishing incident management systems at continental and national levels and embedding Prevention of Sexual Exploitation, Abuse and Harassment (PSEAH) safeguards; risk communication and community engagement (RCCE)—community-centered strategies with social listening and the engagement of traditional healers and faith leaders; surveillance and epidemiology—strengthened Integrated Disease Surveillance and Response (IDSR), exit screening, cross-border information sharing, and contact tracing; laboratory systems and genomic sequencing—deployment of pan-filovirus assays and decentralized sequencing to avoid species-specific detection gaps; case management and clinical care—approximately 15 Ebola Treatment Units (ETUs) and community isolation units, supportive care as the therapeutic mainstay, and a survivor care program; IPC, water, sanitation and hygiene (WASH), and safe and dignified burials—an IPC ring approach, co-designed alternative mourning rituals with communities; research, knowledge management, and access to MCMs—accelerated development of BDBV-specific vaccines and therapeutics through i-MCM-net and clinical trial protocols; operations support, logistics, and workforce deployment—the largest single budget pillar covering pre-positioned stocks, multidisciplinary rapid response teams, and mental health support for responders; and cross-cutting pillars including continuity of essential health services, AI analytics, preparedness and readiness, One Health, humanitarian response, and Prevention of Sexual Exploitation, Abuse and Harassment /safeguarding. The plan is guided by the principles of “One Team, One Plan, One Budget, and One M&E Framework” and coordinated by the IMST co-led by Africa CDC and WHO.^9^

### 5.3 Budget and resource mobilization

The total budget for the Continental Preparedness and Response Plan is approximately $518 million. Of this, roughly $265 million is allocated directly to outbreak response in the Democratic Republic of the Congo and Uganda, while $79 million is earmarked for preparedness in 10 high-risk countries—the Central African Republic receives $16.7 million, Burundi $15 million, South Sudan $7.4 million, and Rwanda, Kenya, Zambia, Tanzania, Ethiopia, Somalia, Angola, and the Republic of Congo $5 million each. A further approximately $174 million represents as-yet unconfirmed partner budgets, which are being coordinated to avoid duplication.^9^

The Africa CDC and the WHO have jointly launched a continental emergency fundraising appeal, while broadening financing channels through weekly donor briefings, investment cases, and private-sector mobilization.^9^ African Union Member States have pledged domestic contributions equivalent to roughly 10% of the total required; the South African government’s early commitment of $5 million was among the first bilateral funds to be secured. Nevertheless, the overall funding gap remains significant, and the timely, flexible availability of funds continues to be the critical bottleneck.^9^

### 5.4 African-led science

The continental plan explicitly calls for “African-led science to drive the continental response”.^9^ reflecting a determination that the continent can no longer depend on exogenous priorities, funding, and supply chains to protect its populations.^9^ African-led research priorities span genomic epidemiology—with institutions like INRB-Kinshasa, the Uganda Virus Research Institute (UVRI), and the African Centre of Excellence for Genomics of Infectious Diseases (ACEGID) leading sequencing and analysis.^51^; clinical research—leveraging networks such as African coaLition for Epidemic Research, Response and Training (ALERRT) and Pan-African Network for Rapid Research, Response, Relief and Preparedness for Infectious Disease Epidemics (PANDORA-ID-NET) for BDBV-specific trials.^9^^,^^26^; social science—community engagement research led by African social scientists to counter misinformation and build trust.^53^; implementation science—comparing effectiveness of NPI delivery models in conflict settings.^59^; and knowledge management—establishing repositories for guidelines, protocols, datasets, and lessons learned to preserve institutional memory between outbreaks.^9^

## 6 Conclusions

The 2026 BVD outbreak is only the third recorded Bundibugyo virus epidemic and has become Africa’s largest Ebola event since the 2014–2016 West African crisis. The multi-day diagnostic delay caused by a *Zaire*-specific assay at Bunia epitomizes the *Zaire*-centric blind spot that has left global preparedness systems ill-equipped for non-Zaire filovirus species. The convergence of absent MCMs, armed conflict, and community mistrust has made non-pharmaceutical interventions the absolute mainstay of containment. The attack on a safe burial team and the collapse of hospital attendance starkly illustrate that the effectiveness of any technical intervention is mediated by its social and political context.

The 14-pillar, $517.7 million continental plan co-led by Africa CDC and WHO represents a new paradigm of African public health governance. However, confirmed funding—approximately 10% from African domestic pledges and early bilateral donations such as South Africa’s—falls far short of total requirements, and the timeliness and flexibility of resource mobilization remain critical uncertainties.

In the longer term, the international community must urgently address the *Zaire*-centric imbalance in filovirus preparedness. First, surveillance and diagnostic networks should incorporate pan-filovirus or multiplex assays as the default screening standard, rather than relying on species-specific tests that are prone to false negatives. Second, the research and development pipeline for vaccines and therapeutics must adopt a multi-species approach; the accelerated development of cross-protective or cocktail strategies that cover all known human-pathogenic ebolaviruses should be prioritized. Third, non-pharmaceutical interventions must be tailored to the specific social and cultural contexts of mining communities and conflict zones—co-designing burial protocols with local leaders and ensuring the safety of response personnel are preconditions for success. Finally, global health governance must move from a reactive charity model to a proactive partnership model in which African institutions co-own the agenda for health security.

## 7 Perspectives and recommendations for emerging outbreaks of known pathogens

Beyond the immediate response, the 2026 BVD outbreak offers several transferable lessons for the scientific prevention and control of emerging epidemics caused by known but neglected pathogens.

**From *Zaire*-centric to pan-filovirus diagnostics.** The false-negative GeneXpert result at Bunia was a sentinel event that should trigger a global review of deployed molecular diagnostics in Ebola-prone regions. National and regional reference laboratories should maintain capacity for pan-filovirus real-time polymerase chain reaction and, where feasible, metagenomic sequencing to enable species-agnostic detection.^51^^,^^60^ Multiplex point-of-care tests that simultaneously detect Bundibugyo, Zaire, Sudan, and Marburg viruses are needed to avoid single-species bias in triage settings.

**Accelerating broad-spectrum countermeasures.** The current MCM pipeline, heavily skewed toward EBOV, is ill-suited to an era of serial filovirus emergence. Structure-based immunogen design that targets conserved epitopes across the *Orthoebolavirus* genus.^61^^,^^62^ and the development of antiviral agents targeting the polymerase active site—which is more conserved than the glycoprotein—should receive sustained investment. Ring vaccination trials using multivalent vaccine candidates could be prepared in advance for rapid activation during future non-Zaire outbreaks.

**Conflict-sensitive and community-embedded implementation.** The attack on the SDB team and the collapse of health service utilization in Bunia underscore that even the most evidence-based NPI is unworkable without community trust. Context-adapted strategies—such as hiring local burial workers from affected communities, engaging traditional healers in alert reporting, and embedding psychosocial support into all response pillars—should become standard operating procedure from the first day of an outbreak.

**One Health surveillance at the source.** The clustering of cases around gold mines strongly suggests that land-use change and ecological disruption facilitate spillover. Establishing longitudinal, integrated human–animal–environmental surveillance in mining concessions and other high-risk interfaces could provide early warning of filovirus activity and reduce the detection delay that characterized the current epidemic.^63^

**Sustainable financing for neglected pathogens.** The $517.7 million continental plan, although substantial, remains largely unfunded. Novel financing instruments—such as an African Epidemic Fund triggerable by Africa CDC for non-Zaire filovirus events—could complement traditional donor mechanisms and ensure that response operations are not paralysed by resource uncertainty.

**Scenario-based preparedness and adaptive response.** Looking ahead, the 2026 BVD outbreak may follow one of three plausible trajectories. The most optimistic—rapid containment—hinges on the immediate closure of funding gaps, guaranteed humanitarian access, and a rapid restoration of community trust, all of which remain uncertain as of the latest reporting period. The current trajectory, characterized by sustained geographic expansion along mining corridors and porous border crossings, is more likely to persist unless cross-border coordination is intensified and systematic exit–entry screening is fully operationalized at all major points of entry. A third, more concerning possibility is the endemic establishment of Bundibugyo virus along the gold-mining belt of eastern DRC: Repeated zoonotic spillovers and undetected human-to-human transmission cycles could create a permanent reservoir of infection. To prepare for these eventualities, response infrastructure must be designed for durability rather than acute surge alone. Permanent screening posts at key border crossings, longitudinal “One Health” surveillance sentinel sites co-located with mining concessions, and pre-negotiated protocols for the rapid deployment of investigational vaccines and therapeutics during non-Zaire filovirus events should be established now. The 42-day countdown that conventionally marks the end of an Ebola outbreak must not be misinterpreted as a signal to dismantle capacities, because persistent viral reservoirs in survivors or wildlife could reignite transmission months after the last recognized case. Integrating scenario-based preparedness into national and continental planning will allow the global community to pivot from reactive containment to proactive risk management, not only for Bundibugyo virus but also for other neglected filovirus species with epidemic potential.

## CRediT authorship contribution statement

**Yi Zhang:** Writing – original draft, Methodology, Conceptualization. **Junkai Ren:** Writing – original draft, Data curation. **Xuejun Ma:** Writing – original draft, Data curation. **Xiaozhou He:** Writing – review & editing.

## Informed consent

Not applicable.

## Organ donation

Not applicable.

## Ethical statement

Ethics approval was waived for this study because no patients’ data were reported.

## Animal treatment

Not applicable.

## Generative AI

Not applicable.

## Funding

This research was funded by the National Key Research and Development Program (grant number 2024YFC2311502 and 2024YFC2310901) and the Young Scholar Scientific Research Foundation of IVDC, China CDC (IVDC-202404).

## Declaration of competing interest

The authors declare that they have no known competing financial interests or personal relationships that could have appeared to influence the work reported in this paper.

## Data Availability

Data sharing is not applicable to this article as no data sets were generated or analyzed during the current study.
